# Drug Abuse: Meth’s Pollution Epidemic

**DOI:** 10.1289/ehp.113-a589a

**Published:** 2005-09

**Authors:** Carol Potera

Methamphetamine problems are soaring nationwide. Nearly 60% of county officials report that meth is the largest drug problem in their county, and 87% saw jumps in meth arrests in the past three years, according to a survey released in July 2005 by the National Association of Counties. But meth isn’t just a hard drug; it’s also an environmental hazard.

Illicit drug makers can cook small batches of meth anywhere they can plug in a stove, microwave, or electric skillet (heat is not required, but speeds the manufacture). The ingredients include common cold medicines, ammonia fertilizer, and muriatic acid. Cooking generates a variety of noxious solvents and gases, such as hydrogen chloride, phosphine, and meth itself. According to an 8 August 2005 *Newsweek* article, for each pound of meth produced, five pounds of toxic waste are left behind.

Police and firemen report breathing problems and headaches when they bust meth labs, but no one has quantified the hazards they face. So John Martyny, an industrial hygienist at National Jewish Medical and Research Center in Denver, teamed up with law enforcement officials in Colorado. They set up controlled cooks in an abandoned motel (which was later razed) and measured the resulting pollutants.

In the unpublished studies, phosphine gas reached 2.9 parts per million (ppm), three times the occupational short-term exposure limit. Phosphine causes headache, pulmonary edema, and death. Martyny says cook fatalities are probably linked to this chemical. Hydrogen chloride fumes reached 155 ppm, more than three times the level considered by the National Institute for Occupational Safety and Health to be “immediately dangerous to life or health.” Hydrogen chloride causes respiratory tract damage. Ammonia, which causes lung edema, also soared to three times the “immediately dangerous to life or health” level.

Anyone who is present during cooks is exposed to these and likely other toxicants; a third of all meth busts find children present. “The health costs to children may not be identified for years to come,” says Martyny, who predicts long-term respiratory and neurological problems.

More research is needed to determine the best ways to clean up meth labs. Meth becomes airborne during production and settles on surfaces at up to 16,000 micrograms per 100 square centimeters (μg/100 cm^2^). Even six months after a staged cook, Martyny found meth levels of 300 μg/100 cm^2^ on surfaces. Carpets trap meth and other pollutants, yet vacuuming dra-meth levels. So Martyny recommends discarding carpets.

Further, after grinding contaminated wallboard in separate unpublished studies, Stephen Lee, who supervises the Emergency Response Team at the Minnesota Pollution Control Agency in St. Paul, learned that washing walls removes less than 10% of the total meth. The rest is trapped deeper. Whether it bleeds back out to the surface and poses an exposure risk is unknown. Lee is evaluating whether a covering of oil-based paint seals meth within wallboard.

Few states have guidelines for cleaning up meth labs. “As more states deal with remediation of meth properties, they turn to us,” says Carolyn Comeau, manager of the Clandestine Drug Lab Program at the Washington State Department of Health. Washington requires remediation by contractors certified by the state to decontaminate meth labs, which are often found in low-income rental properties. The state also requires that surface meth be at or below 0.1 μg/100 cm^2^ before new residents can move in. Scientific evidence like Martyny’s and Lee’s should yield more effective guidelines, says Comeau.

Lee adds that a health-based standard for meth residues on building surfaces is needed to determine which properties need remediation and when a property has been adequately cleaned. All three experts support national remediation standards like those proposed in the Methamphetamine Remediation Research Act of 2005, which would establish a federal research program at the Environmental Protection Agency to study the environmental and health effects of meth labs and coordinate cleanup efforts. The bill has 55 cosponsors, and floor passage in the House is expected later this year.

## Figures and Tables

**Figure:**
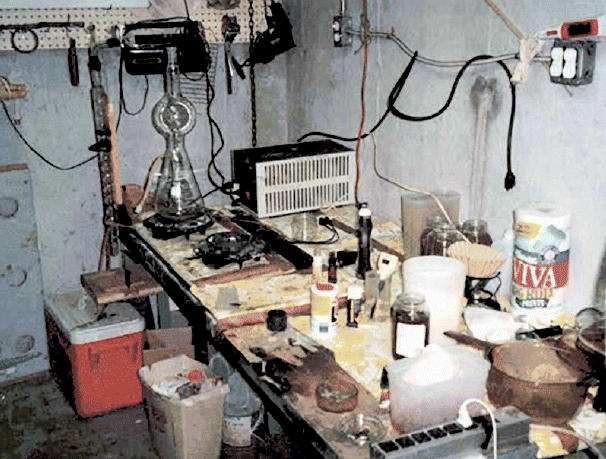
Basement time-bomb. A home meth lab produces toxic waste along with illegal drugs.

